# Probing the ATP Site of GRP78 with Nucleotide Triphosphate Analogs

**DOI:** 10.1371/journal.pone.0154862

**Published:** 2016-05-04

**Authors:** Scott J. Hughes, Tetyana Antoshchenko, Yun Chen, Hua Lu, Juan C. Pizarro, Hee-Won Park

**Affiliations:** 1 Department of Pharmacology and Toxicology, University of Toronto, Toronto, Ontario M5G 1L7, Canada; 2 Department of Biochemistry and Molecular Biology, Tulane School of Medicine, New Orleans, LA 70112, United States of America; 3 Department of Tropical Medicine, Tulane University School of Public Health and Tropical Medicine, New Orleans, LA 70112, United States of America; Weizmann Institute of Science, ISRAEL

## Abstract

GRP78, a member of the ER stress protein family, can relocate to the surface of cancer cells, playing key roles in promoting cell proliferation and metastasis. GRP78 consists of two major functional domains: the ATPase and protein/peptide-binding domains. The protein/peptide-binding domain of cell-surface GRP78 has served as a novel functional receptor for delivering cytotoxic agents (e.g., a apoptosis-inducing peptide or taxol) across the cell membrane. Here, we report our study on the ATPase domain of GRP78 (GRP78_ATPase_), whose potential as a transmembrane delivery system of cytotoxic agents (e.g., ATP-based nucleotide triphosphate analogs) remains unexploited. As the binding of ligands (ATP analogs) to a receptor (GRP78_ATPase_) is a pre-requisite for internalization, we determined the binding affinities and modes of GRP78_ATPase_ for ADP, ATP and several ATP analogs using surface plasmon resonance and x-ray crystallography. The tested ATP analogs contain one of the following modifications: the nitrogen at the adenine ring 7-position to a carbon atom (7-deazaATP), the oxygen at the β-γ bridge position to a carbon atom (AMPPCP), or the removal of the 2’-OH group (2’-deoxyATP). We found that 7-deazaATP displays an affinity and a binding mode that resemble those of ATP regardless of magnesium ion (Mg^++^) concentration, suggesting that GRP78 is tolerant to modifications at the 7-position. By comparison, AMPPCP’s binding affinity was lower than ATP and Mg^++^-dependent, as the removal of Mg^++^ nearly abolished binding to GRP78_ATPase_. The AMPPCP-Mg^++^ structure showed evidence for the critical role of Mg^++^ in AMPPCP binding affinity, suggesting that while GRP78 is sensitive to modifications at the β-γ bridge position, these can be tolerated in the presence of Mg^++^. Furthermore, 2’-deoxyATP’s binding affinity was significantly lower than those for all other nucleotides tested, even in the presence of Mg^++^. The 2’-deoxyATP structure showed the conformation of the bound nucleotide flipped out of the active site, explaining the low affinity binding to GRP78 and suggesting that the 2’-OH group is essential for the high affinity binding to GRP78. Together, our results demonstrate that GRP78_ATPase_ possesses nucleotide specificity more relaxed than previously anticipated and can tolerate certain modifications to the nucleobase 7-position and, to a lesser extent, the β-γ bridging atom, thereby providing a possible atomic mechanism underlying the transmembrane transport of the ATP analogs.

## Introduction

Nucleoside analogs have been in clinical use for almost 50 years and are considered cornerstones of treatment for patients with cancer or viral infections [1]. For instance, FDA-approved nucleoside analogs are used for the treatment of hematological malignancies and, to a lesser extent, solid tumors (www.drugbank.ca). The nucleoside analogs are prodrugs that require biotransformation to the active drug compounds (i.e., an addition of three phosphates to nucleoside analogs that produce nucleotide triphosphates (NTPs)) by intracellular kinases after entering cells via nucleoside transporters. Unfortunately, the higher frequency of mutations in cancer cells, specifically those that alter the activities of prodrug transporters and intracellular activation enzymes, often results in resistance to nucleoside analogs [2–4]. A simple solution for this resistance to nucleoside analogs is to administer NTP analogs that can enter cells independent of nucleoside transporters and do not require intracellular kinases for activation. However, relatively little attention has been paid to NTP analogs as a drug platform primarily due to their poor permeability across cell membrane.

Cell-surface GRP78 is an excellent candidate for a cancer-specific intracellular delivery system of NTP analogs, particularly ATP analogs, for several reasons as follows. First, there is evidence for the relocation of GRP78 from the ER to the cell surface in numerous cancer cells, where it has roles in promoting cell proliferation and metastasis ([5] and references therein). This evidence suggests that cell-surface GRP78 can be targeted for delivering ATP analogs into cancer cells. Second, GRP78 is typically absent on the cell surface of normal cell lines and major adult organs [6]. This finding suggests that the GRP78-targeted ATP analogs would have minimal nonspecific toxicity toward normal tissues, thereby eliminating potential side effects and promoting their clinical impact. Third, engineered agents that fuse a cytotoxic agent (e.g., a apoptosis-inducing peptide or taxol) with a peptide specific for the protein/peptide-binding domain of GRP78 can bind to cell-surface GRP78, become internalized, and cause cancer cell death [7–9]. This finding raises the possibility that, similar to the protein/peptide binding domain, the ATPase domain of cell-surface GRP78 can be developed as a novel functional receptor for delivering ATP analogs across the cell membrane.

To serve as an efficient transmembrane delivery system of ATP analogs, GRP78_ATPase_ needs to have relaxed nucleotide specificity so as to tolerate a wide range of ATP modifications. Previous studies with AMPPNP and ATPγS, which contain substitutions at the β-γ bridging and γ non-bridging oxygen atoms, respectively [10–12], have shown that GRP78_ATPase_ can tolerate modifications in the β-γ bridging and the γ non-bridging positions of ATP, albeit with penalty of low binding affinity. However, very little attention has been devoted to determine the effects of ribose sugar and nucleobase modifications on the binding to GRP78_ATPase_. Information regarding their binding properties would be of value in designing the highest affinity ATP analogs. Therefore, we studied the ability of GRP78_ATPase_ to interact with three ATP analogs (i.e., 7-deazaATP, AMPPCP and 2’-deoxyATP) and the known substrate/product (i.e., ATP and ADP) by using surface plasmon resonance and x-ray crystallography. Our results showed that these ATP analogs do indeed display different affinities to GRP78_ATPase_, as detailed below. While 7-deazaATP bound to GRP78_ATPase_ with a similar binding affinity as ATP and ADP, AMPPCP showed a reduced binding affinity to GRP78_ATPase_, likely due to the replacement of the oxygen at the triphosphate β-γ bridge with a carbon atom. The crystal structures of 7-deazaATP and AMPPCP, compared to those of ATP and ADP, showed that they bind to GRP78_ATPase_ in a manner similar to the endogenous substrate/product. Conversely, the removal of the 2’-OH group of ATP (2’-deoxyATP) caused a marked reduction in the binding affinity to GRP78_ATPase_. Structural analysis revealed that 2’-deoxyATP binds to GRP78_ATPase_ through a unique binding mode: the ribose sugar and triphosphate are flipped out of the active site while the adenine ring is still bound at the nucleobase-binding pocket. Taken together, these results demonstrate various nucleotide analog-GRP78_ATPase_ interactions at an atomic level within the nucleotide-binding active site and provide structural information for improving the properties of next generation of ATP analogs.

## Materials and Methods

### Protein Purification

GRP78_ATPase_ (residues 26–407) was amplified from the full-length human GRP78 (residues 1–654) by PCR, ligated into a pET28-MHL expression vector containing an N-terminal hexahistidine tag (GenBank accession EF456735), and subsequently transformed into *E*.*coli* BL-21(DE3) (Stratagene) competent cells. Successful transformants were grown in Luria Bertani broth (Sigma) for 16 hrs at 37°C, and then transferred to Terrific Broth (Sigma). Growth at 37°C continued until the OD_600_ reached ~3.0, whereupon the temperature was lowered to 18°C and protein expression was induced by the addition of 0.1 mM isopropyl β-D-thiogalactopyranoside. Sixteen hours post-induction, cells were collected by centrifugation and resuspended in binding buffer (30 mM Tris-HCl pH 7.5, 200 mM NaCl, 1 mM β-ME) supplemented with 0.1% octylphenoxypolyethoxyethanol, 1 mM benzamidine, and 1 mM phenylmethanesulfonyl fluoride. Cells were lysed by French press, and the lysates were clarified by centrifugation. The supernatant was loaded on to a nickel-nitrilotriacetic acid column pre-equilibrated with binding buffer. Following a wash step with binding buffer containing 30 mM imidazole, the bound protein was eluted using a linear gradient from 30 to 500 mM imidazole. The eluate was then loaded on to a 26/60 Superdex 200 size exclusion column (GE Healthcare) pre-equilibrated with 30 mM Tris-HCl pH 7.5, 200 mM NaCl and 1 mM β-ME. Target protein-containing fractions were pooled and concentrated to 30 mg/ml using 10-kDa centrifugal filter units (Amicon), and then frozen in aliquots at -80°C until further use

### Crystallization, Data Collection and Structure Solution

**Fig 1:**
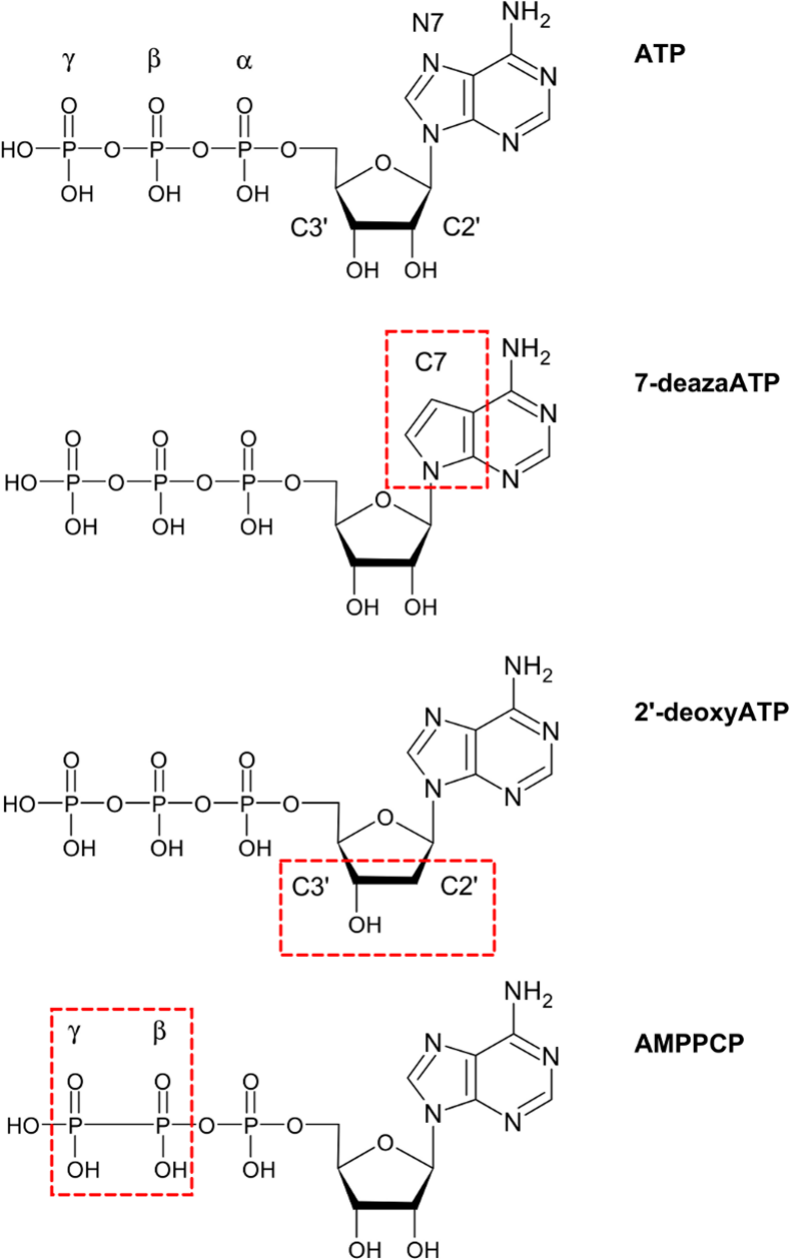
Chemical structures of the ATP analogs used in this study. The structures of ATP, 7-deazaATP, 2'-deoxyATP and AMPPCP with key atom positions indicated. Boxes denote the region—nucleobase, sugar, triphosphate—containing the modification for that analog.

Crystallization trials were conducted using the sitting-drop, vapor diffusion method and in-house screening kits, with each drop containing a 1:1 ratio of purified GRP78_ATPase_ and reservoir solution. Drops were set in 96 well Intelli-Plates (Hampton Research) and incubated at room temperature until crystal growth was observed. Initially the triclinic crystals of apo GRP78_ATPase_ were observed in 0.1 M Tris- HCl pH 8.5, 25% PEG 3350 and 0.1 M Na/K tartrate. Despite crystals diffracting to ~2 Å, crystal structure analysis indicated that packing-induced blockage of the active site rendered the crystals not suitable for soaking experiments, as the crystal packing was similar to the previously reported triclinic crystals of GRP78_ATPase_ [13]. However, microseeding with the initial crystals yielded a "soakable" monoclinic crystal form in a new condition (24–26% PEG 3350, 0.1 M Tris-HCl and 0.2 M NaCl), with the crystal packing similar to the previously reported monoclinic crystals of GRP78_ATPase_ [12]. These new apo monoclinic crystals were soaked with either 10 mM ATP (Sigma), 5 mM ADP (Sigma), 10 mM AMPPCP, 5 mM 7-deazaATP, or 25 mM 2'-deoxyATP overnight (one week for 7-deazaATP) at room temperature. The chemical structures of ATP and each ATP analog can be found in Fig 1. Note that Mg^++^ was excluded in crystallization and soaking solutions. Crystals were cryoprotected in an equal (1:1) mixture of paratone-N and mineral oil and flash-frozen in liquid nitrogen until data collection.

Data was collected at the Canadian Light Source 08ID-1 beamline in Saskatoon, Canada, as well as the Advanced Photon Source 23-IDB and 19-ID beamlines at the Argonne National Laboratory in Chicago, Illinois. ATP- and 7-deazaATP-bound GRP78_ATPase_ data were processed using the programs XDS [14] and SCALA [15], while the ADP-, AMPPCP- and 2'-deoxyATP-bound GRP78_ATPase_ data processing was carried out using the software suite HKL2000 [16]. All structures were solved by molecular replacement via the program MOLREP [17] in the CCP4 crystallographic suite [18]. The 7-deazaATP structure was solved using the ligand-free GRP78_ATPase_ structure (PDB ID: 3LDN) as a search model; all subsequent solutions used this 7-deazaATP structure. After molecular replacement, each model was subjected to a single round of simulated annealing with CNS Solve [19], followed by multiple rounds of model building and refinement using Coot [20] and REFMAC [21], respectively. All riding hydrogens were excluded from the output coordinate files but included for refinement. 7-deazaATP restraints for refinement were generated and optimized using the programs PRODRG [22] and eLBOW [23]. Model geometry was validated using MolProbity [24], while TLS parameters were validated using TLSANL [25]. Coordinates for all five GRP78_ATPase_ structures have been deposited in the Protein Data Bank with accession codes: 5EVZ (ADP + P_i_), 5F1X (ATP), 5EXW (7-deazaATP), 5EY4 (2'-deoxyATP), 5F2R (AMPPCP). Summary data collection and refinement statistics can be found in Table 1. Figures were generated using the PyMOL Molecular Graphics System (Version 1.5.0.4 Schrödinger, LLC).

**Table 1:** Data collection and refinement statistics.

Ligand	*ATP*	*ADP*	*AMPPCP*	*7-deazaATP*	*2'-deoxyATP*
PDB ID	5F1X	5EVZ	5F2R	5EXW	5EY4
Data Collection	23-IDB APS	08ID-1 CLS	08ID-1 CLS	08ID-1 CLS	19-ID APS
X-ray source					
Wavelength (Å)	1.0332	0.97949	0.97949	0.97949	0.97918
Resolution (Å)	44.5–1.90	44.3–1.82	49.5–2.10	44.3–1.90	34.2–1.86
Space Group	P2_1_	P2_1_	P2_1_	P2_1_	P2_1_
No. of molecules in ASU	2	2	2	2	2
Unit cell parameters (Å,°)	a = 56.0, b = 74.8, c = 90.2, β = 98.9	a = 55.6, b = 74.8, c = 89.2, β = 98.6	a = 55.0, b = 74.5, c = 88.4, β = 98.2	a = 55.7, b = 74.8, c = 89.7, β = 98.8	a = 56.5, b = 74.8, c = 85.7, β = 98.3
No. of unique reflections^a^	58009 (8461)	64199 (3210)	40808 (2041)	57406 (8337)	59159 (2958)
Completeness (%)	99.9 (100.0)	99.9 (99.9)	99.3 (95.9)	100.0 (100.0)	99.7 (98.5)
Wilson B-Factor (Å^2^)	21.7	23.1	27.0	17.4	22.8
Friedel Redundancy	3.8 (3.8)	5.0 (4.6)	4 (3.1)	4.9 (5.0)	4.5 (3.7)
R_merge_ (%)^b^	11.2 (65.4)	7.7 (45.7)	13.2 (40.9)	5.5 (17.6)	5.9 (21.6)
Average I/σ	8.3 (2.2)	22.6 (5.3)	15.7 (4.3)	19.1 (9.5)	28.3 (7.2)
Refinement					
Resolution (Å)	44.5–1.90	44.3–1.85	44.0–2.15	40.0–1.90	34.0–1.86
Completeness (%)	99.7	99.8	99.3	99.8	99.3
R_work_/R_free_ (%)^c^	18.3/22.8	16.5/20.5	19.9/23.4	16.5/20.8	17.3/21.2
No. of atoms/ Average B-factors (Å^2^)					
Protein	5905/ 31.9	5920/ 32.2	5900/ 35.9	5905/ 27.2	5899/ 26.6
Ligand/ion	62/ 19.5	66/ 19.9	64/ 25.3	62/ 16.2	60/ 42.7
Water	429/ 29.1	534/ 31.7	221/ 27.9	653/ 30.9	510/ 30.7
RMSD bond length (Å)	0.011	0.008	0.013	0.010	0.010
RMSD bond angle (°)	1.273	1.34	1.274	1.177	1.226
Ramachandran Analysis					
Favored (%)	99.2	98.6	98.3	99.2	99.1
Outliers (%)	0	0	0	0	0

^*a*^ Numbers in parentheses are for the outer shell.

^b^ *R*_merge_ = Σ[(*I* − <*I*>)]*/*Σ(*I*), where *I* is the observed intensity and *<I>* is the average intensity.

^c^ *R*_work_ = Σ[|*F*_obs_| − |*F*_calc_|]/Σ|*F*_obs_|, where |*F*_obs_| and |*F*_calc_| are magnitudes of observed and calculated structure factors respectively. R_free_ was calculated as R_work_ using 5.0% of the data, which was set aside for an unbiased test of the progress of refinement.

### Surface Plasmon Resonance (SPR)

SPR measurements were performed on Biacore T200 instrument (GE Healthcare) at 25˚C. Purified GRP78_ATPase_ was immobilized on a CM5 sensorchip using NHS/EDC coupling following the manufacturer protocol to a level of ~7000 RUs, a reference surfaces without immobilized GRP78_ATPase_ served as a control for nonspecific binding and refractive index changes. Five different concentrations of the ligands (0.01, 0.4, 1.5, 6.25 and 25 μM for ADP and ATP; 0.04, 0.15, 0.625, 2.5 and 10 for 7-deazaATP; 12.5, 25, 50, 100 and 200 μM for AMPPCP; 31.25, 62.5, 125, 250 and 500μM for 2'-deoxyATP) were injected in triplicate over the sensor chip at 30 μL/min in random order. Buffer alone injections were used as blanks. The sensor surface was not regenerated between injections but a 10 min long dissociation step was sufficient to recover baseline levels. The running buffer was 10 mM HEPES, pH 7.4, 150 mM NaCl, 0.005% P20 and 2 mM MgCl_2_. Biacore equilibrium responses recorded at the end of the association step were used to determine the dissociation constant (K_d_). Nonspecific signal binding was removed by subtracting a reference channel without GRP78_ATPase_. Binding responses at the different concentrations were globally fitted to a saturation-binding model in Prism 6 (GraphPad Software Inc., California).

The effect of Mg^++^ on binding affinities was explored by repeating the same SPR protocol with two additional running buffers. The first running buffer contained no addition of Mg^++^ to determine the effect of Mg^++^ co-purified with GRP78_ATPase_ (10 mM HEPES, pH 7.4, 150 mM NaCl and 0.005% P20), and the second running buffer contained 5 mM EDTA to chelate any Mg^++^ present in the system (10 mM HEPES, pH 7.4, 150 mM NaCl, 0.005% P20 and 5 mM EDTA).

## Results

### ADP and ATP binding to the ATPase domain of GRP78 (GRP78_ATPase_)

**Fig 2:**
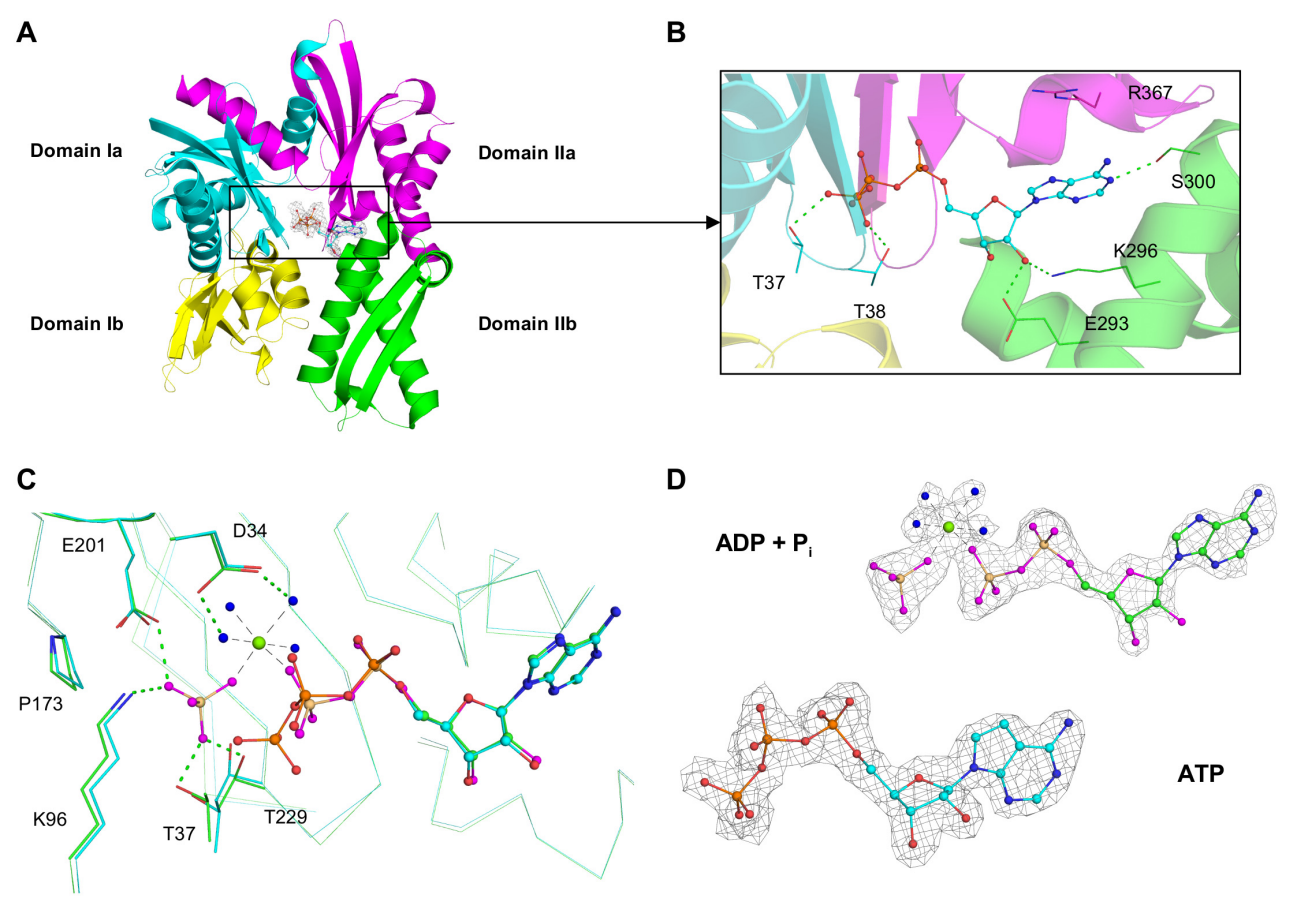
GRP78_ATPase_ domain structure and canonical ATP/ADP binding site. **(A).** Overview of the GRP78_ATPase_ subdomain structure: Ia (cyan), Ib (yellow), IIa (purple), and IIb (green). **(B).** Close-up of ATP binding site indicates key interacting residues. **(C)**. Overlay of the ATP (cyan) and ADP-P_i_-Mg^++^ (green) binding sites highlights the difference between the ATP γ-phosphate and P_i_. **(D)**. Fourier difference maps (F_o_-F_c_) of ATP and ADP-P_i_-Mg^++^ contoured to 2.5 σ. Dashed lines indicate potential hydrogen bonds (green) and metal coordination (black). Non-bonding spheres indicate water (blue) and magnesium (green). For clarity, the oxygen atoms of ADP are shown in magenta.

To confirm that we can introduce the substrate ATP and its hydrolysis product ADP into the crystalline form of GRP78_ATPase_ by using a soaking strategy, we grew the crystals of GRP78_ATPase_ in the absence of the nucleotide (nucleotide-free crystals), and then either ATP or ADP was soaked into the nucleotide-free crystals. Unexpectedly, soaking the crystals with ADP resulted in the active site containing electron density for an inorganic phosphate (P_i_) and Mg^++^ in addition to ADP (Fig 2). A possible explanation for this observation is that P_i_ and Mg^++^ were co-purified from recombinant protein expression *E*. *coli* cells and co-crystallized with GRP78_ATPase_. Since there is no GRP78_ATPase_ structure in complex with the ATP hydrolysis products (ADP, P_i_ and Mg^++^), we compared our structure to the ATPase domains of two human Hsp70 proteins, Hsp70-1 and Hsp70-2, that contain the ATP hydrolysis products at the active site [13]. While overall structural similarities were low (Cα-RMSD values of 7.8 Å and 6.4 Å to Hsp70-1 and Hsp70-2, respectively), the positions and orientations of the ATP hydrolysis products in GRP78_ATPase_ matched well with those in the ATPase domains of Hsp70-1 and Hsp70-2. Therefore, the ADP structure of GRP78_ATPase_ appears equivalent to the structure of ATP hydrolysis products and will be referred to as the ADP-P_i_-Mg^++^ product structure. By comparison, ATP soaking resulted in no electron density for P_i_ and Mg^++^ at the active site of the ATP structure (Fig 2). We speculate that the binding of a new ATP molecule to the active site releases the pre-bound P_i_, thereby allowing the pre-bound Mg^++^ to promote ATP hydrolysis; the subsequent product release would then empty the active site. Since present in an excess amount, a second ATP molecule could bind to the now empty active site of GRP78_ATPase_ and remain bound without being hydrolyzed. The observed ATP conformation resembles that of the previous ATP structure of GRP78_ATPase_ (PDB code 3LDL; [12]) (a Cα-RMSD value of 0.34 Å), and this structure will hereafter be called the metal-free ATP structure. Taken together, these results confirm our ability to soak the nucleotides into pre-grown GRP78_ATPase_ crystals without disrupting the crystal lattices.

Superposition of the metal-free ATP and the ADP-P_i_-Mg^++^ product structures shows the γ-phosphate of metal-free ATP is found ~3.4 Å away from P_i_ in the ADP-P_i_-Mg^++^ product structure (Fig 2C) (an Cα-RMSD value of 0.43 Å). The γ-phosphate forms hydrogen bonds with Thr38 and Thr39 from the β1-β2 loop and Gly228 and Thr229 from the β11-β12 loop. This may reduce fluctuations in the terminal γ-phosphate, thereby conferring stability to the bound metal-free ATP. Alternatively, the P_i_ in the ADP-P_i_-Mg^++^ product structure, while still coordinated to the bound Mg^++^, is located in a pocket formed by Thr37, Lys96, Glu201, and Thr229. The γ-phosphate of metal-free ATP is in a distal position with respect to Mg^++^ in the ADP-P_i_-Mg^++^ complex, probably because it would lose many direct hydrogen bonds with the protein residues if proximally oriented in the absence of Mg^++^. This finding provides a structural basis for how the γ-phosphate prefers the distal position when metal-free ATP binds at the active site.

The interactions of ATP and ADP with GRP78_ATPase_ can be described in terms of the strength of their binding affinities. Surface plasmon resonance (SPR) was used to determine the dissociation constant (K_d_) for each nucleotide-GRP78_ATPase_ interaction. Measurements were also taken in the presence (2 mM MgCl_2_) and absence (5 mM EDTA) of Mg^++^, and the corresponding K_d_ values were compared to the untreated condition. The results showed that ATP interacts strongly with purified GRP78_ATPase_ in the untreated condition, while the addition or removal of Mg^++^ minimally affected the high binding affinity of ATP to GRP78_ATPase_ (e.g., K_d_ values in the low micromolar to high nanomolar ranges) (Table 2). The ADP-GRP78_ATPase_ interaction was as strong as the ATP-GRP78_ATPase_ interaction in the untreated condition, as indicated by their similar K_d_ values. However, the ADP binding affinities were significantly different upon the addition (23-fold greater) and removal (159-fold lower) of Mg^++^. Therefore, the ATP-GRP78_ATPase_ interaction seems to be insensitive to Mg^++^ concentration, whereas the ADP-GRP78_ATPase_ interaction depends on Mg^++^ presence at the active site. Combined with the above crystallographic data, the binding data provide a clear basis for the compatible tight binding of ATP and ADP to GRP78_ATPase_.

**Table 2:** GRP78 _ATPase_ nucleotide affinities (K_d_) determined by SPR.

		K_d_ (M)^a^	
SPR running buffer	2 mM MgCl_2_	Untreated^b^	5 mM EDTA
ATP	(4.5 ± 2.9) x 10^−7^	(7.8 ± 7.1) x 10^−7^	(9.8 ± 4.4) x 10^−6^
ADP	(1.2 ± 0.9) x 10^−8^	(2.7 ± 4.4) x 10^−7^	(4.3 ± 7.7) x 10^−5^
7-deazaATP	(3.0 ± 2.0) x 10^−8^	(1.5 ± 0.9) x 10^−7^	(9.0 ± 5.4) x 10^−7^
AMPPCP	(5.9 ± 1.2) x 10^−5^	(5.2 ± 4.1) x 10^−5^	>1 x 10^−3^
2'-deoxyATP	N/A	(7.5 ± 5.0) x 10^−4^	>1 x 10^−3^

^a^ K_d_ values are presented as mean ± SD of three independent experiments (n = 3)

^b^ Untreated denotes that SPR running buffer contains no addition of either MgCl_2_ or EDTA.

N/A = Binding data did not allow a reliable estimation of K_d_.

### AMPPCP binding to GRP78_ATPase_

**Fig 3:**
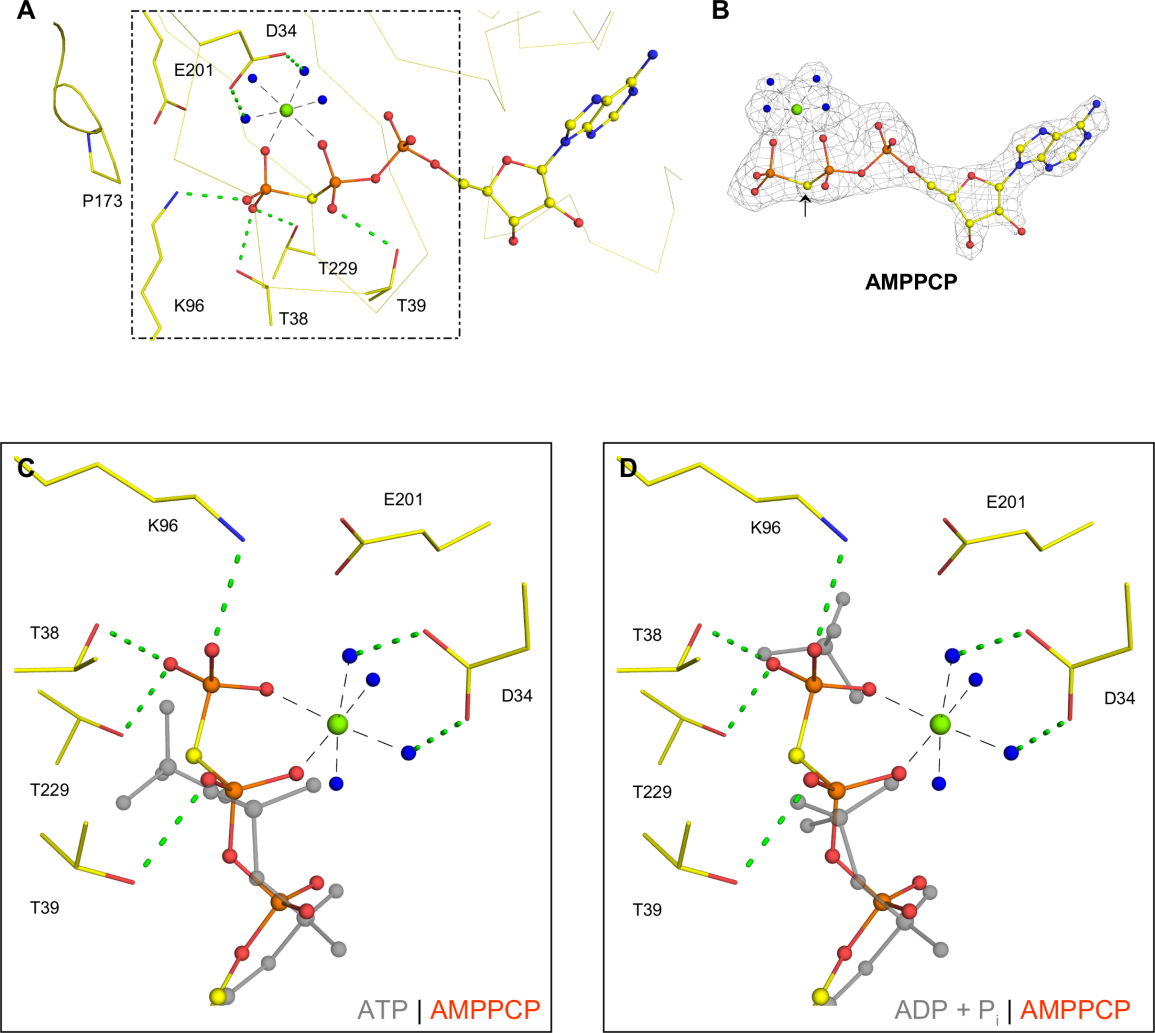
Binding site of AMPPCP. **(A).** The binding site of AMPPCP-Mg^++^-bound (yellow) GRP78_ATPase_ illustrates the key residues interacting with the phosphate groups. Asp224, which interacts with the other Mg^++^-coordinated waters, has been removed for clarity. Dashed lines indicate potential hydrogen bonds (green) and metal coordination (black). Non-bonding spheres indicate water (blue) and magnesium (green; from AMPPCP-Mg^++^). **(B).** Fourier difference map (F_o_-F_c_) of AMPPCP-Mg^++^ contoured to 3.0 σ; the arrow denotes the carbon bridging the β- and γ-phosphates. **(C).** Superposition of α-, β- and γ-phosphate groups in ATP (gray) and AMPPCP-Mg^++^ (orange and red). Residues correspond to the AMPPCP-Mg^++^-bound structure at the active site of GRP78_ATPase_. **(D)**. Superposition of α-, β- and γ-phosphate/P_i_ groups in ADP-P_i_ (gray) and AMPPCP-Mg^++^ (orange and red) at the active site of GRP78_ATPase_. Residues correspond to the AMPPCP-Mg^++^-bound structure.

To determine the effect of modifications at the β-γ bridging oxygen of ATP on GRP78_ATPase_ binding, we performed SPR and crystallographic experiments with AMPPCP, a non-hydrolyzable analog of ATP with the bridging oxygen substituted with a methylene group (CH_2_). SPR measurement showed that AMPPCP displayed poor binding to purified GRP78_ATPase_, with an affinity two-orders of magnitude lower than ATP (Table 2). The addition of 2 mM Mg^++^ did not improve the binding affinity to GRP78_ATPase_, whereas the removal of Mg^++^ by adding 5 mM EDTA almost abolished binding to GRP78_ATPase_. For crystallographic analysis, AMPPCP soaking resulted in electron density at the active site corresponding to AMPPCP and Mg^++^ (Fig 3). The bound Mg^++^ at the active site is consistent with the idea that P_i_ and Mg^++^ were protein co-purification artifacts—P_i_ was released by AMPPCP binding, but Mg^++^ stayed bound to non-hydrolyzable AMPPCP.

The structure shows a number of direct and indirect interactions between AMPPCP, the coordination sphere of Mg^++^, and GRP78 _ATPase_. One non-bridging oxygen atom from each of the β- and γ-phosphates and four water molecules are involved in the coordination of Mg^++^; these four water molecules are further hydrogen-bonded to Asp34 and Asp224 (Fig 3). The other side of the β- and γ-phosphates is directly hydrogen-bonded to the side chains of Lys96 and Thr229 and the backbones of Thr38 and Tyr39 (Fig 3). Despite the large number of interactions between AMPPCP-Mg^++^ and GRP78_ATPase_, the measured binding affinity for AMPPCP was significantly lower than that observed for ATP, even in the presence of 2 mM Mg^++^ (Table 2). This lower affinity is surprising given the similarity to the binding mode of metal-free ATP and the large number of nucleotide-GRP78_ATPase_ contacts.

Superposition of the AMPPCP-Mg^++^ and the ADP-P_i_-Mg^++^ product structures (a Cα-RMSD value of 0.30 Å and Fig 3D) shows that the γ-phosphate of AMPPCP is positioned near P_i_ in the ADP-P_i_-Mg^++^ product structure (~1.0 Å). While this proximal position relative to Mg^++^ occurs because of the interactions between Mg^++^ and the β- and γ-phosphates of AMPPCP, the other side of Mg^++^ is fixed to the surrounding residues through water-mediated hydrogen bonds. Furthermore, superposition of the AMPPCP-Mg^++^ and the metal-free ATP structures (a Cα-RMSD value of 0.56 Å and Fig 3C) shows a clear difference in the γ-phosphate position between the two structures. The imaginary interaction between the γ-phosphate of metal-free ATP and the Mg^++^ of the AMPPCP-Mg^++^ complex would require rotation around the β-γ bridging oxygen. These superposition results suggest that the presence of Mg^++^ swings the γ-phosphate of metal-free ATP from the distal to proximal positions, allowing coordination with Mg^++^ and resulting in the promotion of ATP hydrolysis.

### 7-deazaATP binding to GRP78_ATPase_

To assess the effect of nucleobase modifications on GRP78_ATPase_ binding, we performed SPR and crystallographic experiments with 7-deazaATP, which has the nitrogen at the 7-position replaced with a carbon. SPR measurement showed that the 7-deazaATP affinity for GRP78_ATPase_, regardless of Mg^++^ concentration, closely resembles that of ATP (i.e., the K_d_ values in the low micromolar to high nanomolar ranges; see Table 2). For crystallography, soaking with 7-deazaATP resulted in electron density corresponding to 7-deazaATP, but no density corresponding to P_i_ and Mg^++^ at the active site; this will hereafter be called the metal-free 7-deazaATP structure. Absence of bound P_i_ and Mg^++^ at the active site of GRP78_ATPase_ is likely to be the consequence of excess 7-deazaATP binding to the emptied active site, similar to the metal-free ATP structure (see above).

**Fig 4:**
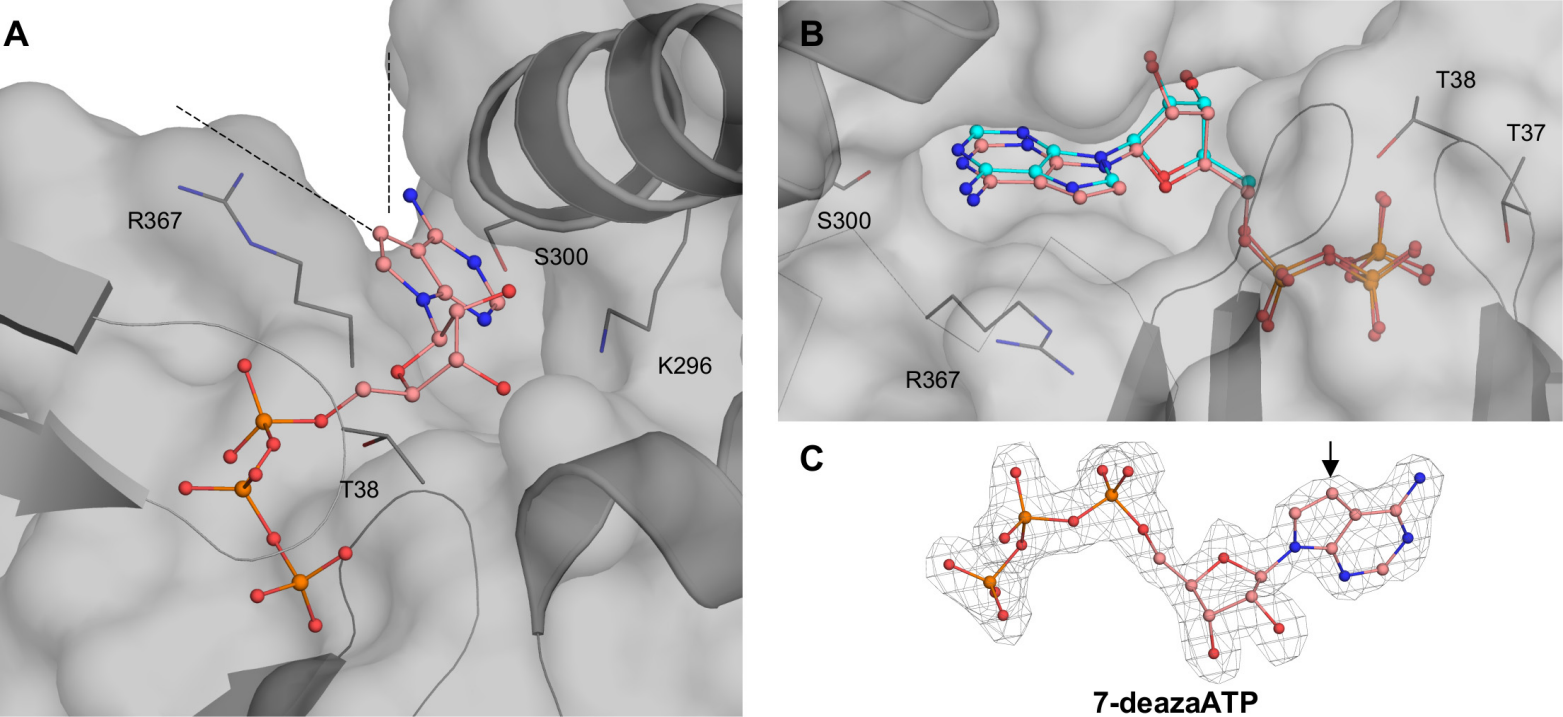
Binding site of 7-deazaATP. (**A)** 7-deazaATP bound to GRP78_ATPase_ illustrates that the 7-position of the adenine ring faces an opening (dash wedge) bordered by Ser300 and Arg367. (**B).** Overlay of ATP ligand (cyan) and 7-deazaATP-bound GRP78_ATPase_ demonstrates the minimal effect of a 7-position modification. (**C).** Fourier difference map (F_o_-F_c_) of 7-deazaATP contoured to 3.0 σ. The arrow denotes the carbon at the 7-position of the adenine ring.

Structural comparison reveals that the protein conformations in the metal-free 7-deazaATP and metal-free ATP structures are very similar to each other, with the bound nucleotides overlapping well in the same binding pocket (a Cα-RMSD value of 0.44 Å and Fig 4B). By examining both structures, we noticed that the atom at 7-position, whether the nitrogen in ATP (N7) or the carbon in 7-deazaATP (C7), makes no significant contact with GRP78_ATPase_ and is exposed to the protein surface (Fig 4A). This finding explains how GRP78_ATPase_ tolerates carbon substitution at N7 of the adenine base of ATP. Moreover, various additional substitutions at the solvent-exposed C7 of 7-deazaATP would unlikely affect the binding affinity to GRP78_ATPase_.

### 2’-deoxyATP binding to GRP78_ATPase_

**Fig 5:**
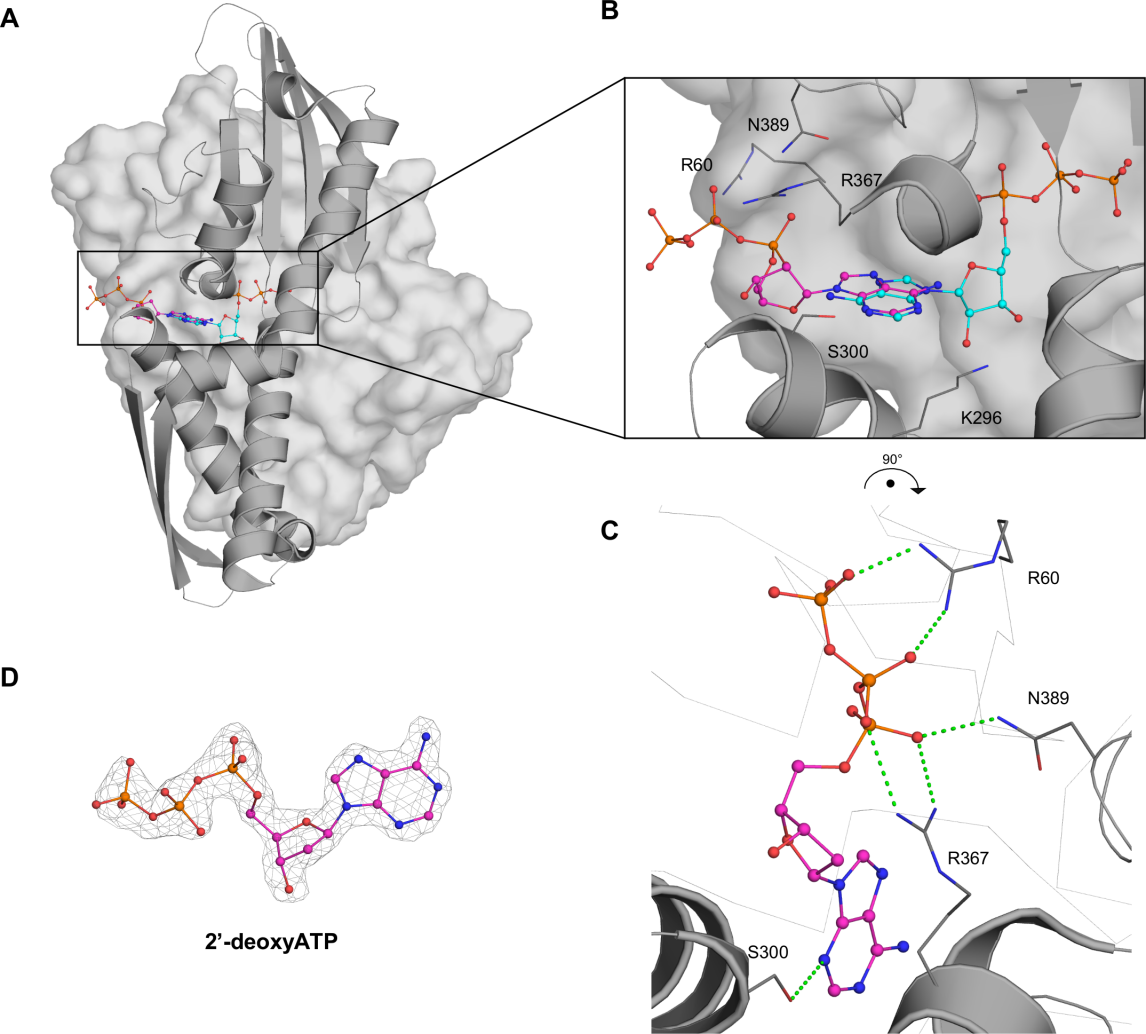
Binding site of 2'-deoxyATP. **(A).** Surface representation of GRP78_ATPase_ shows the outward binding mode of 2'-deoxyATP (magenta) and the inward binding mode of ATP (cyan). **(B)**. Close-up of the 2'-deoxyATP binding site with superimposed ATP (cyan) shows key residues in grey. **(C)**. Alternate view of 2'-deoxyATP binding site highlights interactions with Arg60, Arg367 and Asn389. **(D)**. Fourier difference map (F_o_-F_c_) of 2'-deoxyATP contoured to 3.0 σ. Green dashed lines indicate potential hydrogen bonds.

To evaluate the role and importance of the 2’-OH group of ATP in GRP78_ATPase_ binding, we performed SPR and crystallographic experiments with 2’-deoxyATP, an ATP analog that does not have the 2’ hydroxyl group. Measured by SPR, the K_d_ value of purified GRP78_ATPase_ for 2’-deoxyATP was ~960 times worse than that for ATP in the untreated condition (Table 2). In addition, the removal of Mg^++^ nearly abolished the binding of GRP78_ATPase_ for 2’-deoxyATP (Table 2). The SPR results for 2’-deoxyATP with the addition of 2 mM Mg^++^ were not interpretable, probably due to the presence of two binding modes of GRP78_ATPase_ for 2’-deoxyATP. Comparative analysis revealed that, although similarities between the 2'-deoxyATP and ATP structures were evident (a Cα-RMSD value of 0.80 Å), the 2’-deoxyATP bound to GRP78_ATPase_ with a unique binding mode, having its ribose sugar and triphosphate groups flipped out toward the solvent while the adenine ring remains bound at the nucleobase-binding site (Fig 5). Moreover, the adenine bases of the ATP and 2'-deoxyATP are 180° from each other such that they can be easily superimposed by rotating about an arbitrary axis between the carbon atoms at 2- and 5-positions (C2 and C5). As a result, the amino group at 6-position (C6) is buried in the 2’-deoxyATP structure instead of solvent exposed as in the ATP structure (Fig 5B). Detailed comparison of the binding modes of 2’-deoxyATP and ATP demonstrates that there is an outstanding difference between them in the number of hydrogen bonds with GRP78_ATPase_ residues: only three oxygen atoms of the 2’-deoxyATP triphosphate form direct and water-mediated hydrogen bonds with GRP78_ATPase_ residues (Fig 5C), whereas every oxygen atom of the ATP triphosphate is involved in hydrogen bonding (Fig 2 and data not shown). In addition, the ribose sugar 3’-OH group of 2’-deoxyATP forms water-mediated interactions with Arg297 and Ser301 (Data not shown), whereas both the 2’- and 3’-OH groups of ATP form a network of direct and water-mediated hydrogen bonds with several surrounding residues including Asp259, Glu293 and Lys296 (Fig 2C). These differences in interactions provide an explanation for the lower binding affinity of 2’-deoxyATP relative to ATP. Therefore, the intact ribose sugar is key for sustaining high affinity binding to GRP78_ATPase_.

## Discussion

### Importance of having soakable crystals for ATP analog screening by x-ray crystallography

Cell-surface GRP78, especially the protein-binding domain, has been validated as a functional molecular target/receptor for the transport of cytotoxic agents (e.g., a apoptosis-inducing peptide and taxol) into cancer cells [7] [8] [9]. Advancement in cell-surface GRP78 as a drug delivery receptor opens the door to using the unexploited ATPase domain for the delivery of cytotoxic ATP analogs into cancer cells. A soakable crystal form is an invaluable tool for efficient screening of ATP analogs by x-ray crystallography, as co-crystallization is not readily achievable. A survey of the current literature shows that there are two available crystal forms of GRP78_ATPase_: a triclinic and a monoclinic crystal system [12,13]. Inspection of their crystal packing reveals a solvent channel with access to the nucleotide-binding site in the monoclinic crystals that is absent in the triclinic crystals. In the current work, we have evaluated the monoclinic crystals’ performance as a nucleotide soaking system with ADP, ATP and three different ATP analogs as ligands. The soaking experiments have confirmed the reliability of obtaining the GRP78_ATPase_ structures in various nucleotide-bound states (Figs 2–5). Furthermore, ligand soaking has maintained crystal diffraction quality for molecular replacement phasing and model building, suggesting promise of this approach in future nucleotide-soaking experiments.

### GRP78_ATPase_ serving as an optimal structural scaffold for adenine nucleotide binding

Full-length GRP78 consists of two major functional domains, an ATPase domain and a protein/peptide-binding domain [13]. Our interest lies in determining whether GRP78_ATPase_, represents an ideal structural scaffold for studying the binding affinities and modes of ATP analogs. An overall comparative analysis shows that our ATP-bound GRP78_ATPase_ structure is similar to the corresponding domain of the recent structure of full-length GRP78 bound to ATP (a Cα-RMSD value of 2.57 Å) [26]. A more detailed analysis reveals conformational differences in the localized regions of the ATPase domain (portions of the subdomains Ia, Ib and IIa but not the subdomain IIb) due to the protein/peptide-binding domain only present in the full-length structure, which makes direct contacts to the ATP domain (data not shown). However, these localized structural differences in full-length protein and GRP78_ATPase_ do not change the active site architecture, which has maintained the same binding mode for ATP (data not shown). The common architecture of the active site also predicts that ATP analogs would exhibit similar binding modes in full-length protein and GRP78_ATPase_. Therefore, GRP78_ATPase_ represents a good model system to study ATP analog binding mechanisms to gain knowledge in ATP analog optimization.

### Two different positions of the ATP γ-phosphate in Mg^++^-bound and metal-free states

Superposition of the ADP-P_i_-Mg^++^ structure with the metal-free ATP and AMPPCP-Mg^++^ structures reveals an important role for Mg^++^. The γ-phosphate in the metal-free ATP structure is away (~3.4 Å) from the P_i_ position in the ADP-P_i_-Mg^++^ product structure, a seemingly imperfect orientation for hydrolysis. By comparison, the γ-phosphate in the AMPPCP-Mg^++^ structure is close (~1.0 Å) to the P_i_ position in the ADP-P_i_-Mg^++^ product structure, probably representing the in-line attack conformation [27]. These three structures together suggest that the coordination of Mg^++^ with the β- and γ-phosphates of ATP may induce swinging the γ-phosphate from the distal position to the proximal position for ATP hydrolysis. Thus, the molecular role of Mg^++^ in GRP78 ATP hydrolysis is to induce the movement of γ-phosphate for the phospho-transfer reaction. The requirement of Mg^++^ in GRP78_ATPase_ ATP hydrolysis implies that metal-free ATP can bind to the active site and act as a competitive inhibitor for the ATP-Mg^++^ complex in an excess of ATP over Mg^++^, as previously described [28].

### Importance of 2’-OH group in nucleotide binding to GRP78_ATPase_

We have reported the crystal structure of GRP78_ATPase_ in complex with 2’-deoxyATP that shows it binds to GRP78_ATPase_ with a flipped out conformation (Fig 5), a unique binding mode not shared by other nucleotides (ATP, ADP, 7-deazaATP and AMPPCP), but suffered by a significant loss in binding affinity (Table 2). The ribose sugar 2’-OH group seems to be crucial for the high-affinity binding to GRP78_ATPase_ allowed by a canonical ATP binding mode, as evidenced by the direct and water-mediated hydrogen bonds formed by the 2’-OH group with Glu293 and Lys296 observed in the other nucleotide structures (Figs 2–4) and their low K_d_ values (Table 2). The structure of the complex of the nucleotide exchange factor Sil1 to yeast GRP78_ATPase_ further supports the essentiality of the 2’-OH group in high-affinity binding to GRP78_ATPase_ [29]. The binding of Sil1 changes the conformation of GRP78_ATPase_ to an open state, disrupting those above mentioned hydrogen bonds between the 2’-OH group and Glu293 and Lys296 at the ribose sugar binding site (Fig 2) (Glu313 and Lys316 in yeast protein numbering), as well as another critical interaction between the N1 of adenine and Ser300 at the nucleobase binding site (Fig 2) (Ser320 in yeast protein numbering), thereby causing ADP release from GRP78_ATPase_. This data agrees with an idea that the absence of the 2’-OH group in 2’-deoxyATP disfavors canonical binding at the active site due to the reduction in hydrogen bonding to GRP78_ATPase_. However, the N1-Ser300 interaction allows 2'-deoxyATP to remain bound at the nucleobase-binding site despite being flipped out of the ribosugar and triphosphate binding sites. Taken together, the presence of the 2’-OH group not only contributes to high-affinity binding, but also allows the nucleotide to adopt the canonical binding mode at the active site.

### Potential strategies to improve the metabolic stability and GRP78_ATPase_ selectivity of ATP analogs

In addition to the primary problem of cell permeability, it is known that ATP analogs are readily hydrolyzed by various extracellular nucleotidases (ecto-nucleotidases) [30] and have the potential to activate purinergic P2X and P2Y receptors on the cell surface [31]. These issues may be addressed by upgrading the properties of the ATP analogs, which could then be considered as a drug platform. The upgrade begins by making the ATP analogs resistant to the hydrolysis of ecto-nucleotidases while avoiding their binding to the purinergic receptors and maintaining their high affinity binding to GRP78_ATPase_.

It is evident that most human ecto-nucleotidases (NTPDase1, 2, 3, 8 and NPP1 and 3) cannot hydrolyze an ATP analog containing a carbon dibromide (CBr_2_) bridge between the β- and γ-phosphates (AMPPCBrP) [32]. Our structural investigation with AMPPCP, which contains a CH_2_ group between the β- and γ-phosphates, reveals that this ATP analog shares a similar binding mode to ATP, albeit with a reduced binding affinity (Fig 3 and Table 2). The nucleotide triphosphate binding site contains an extra space for bromine beyond the hydrogen atoms of CH_2_ (Fig 3). These data strengthen an idea that the ATP analog containing the CBr_2_ bridge may behave similar to ATP for the binding to GRP78_ATPase_ but be resistant to ecto-nucleotidase hydrolysis activities.

Analysis of the existing structures of P2X and P2Y receptors (PDB codes 4DW1 and 4PY0) [33,34] show that steric hindrance to the active site residues of the receptors would disallow binding of ATP analogs with substitutions at the nucleobase 7-position. On the contrary, the 7-deazaATP structure reveals that the C7 of the nucleobase is surface-exposed (Fig 4), allowing various substitutions at this position. Thus, 7-substitution may be key to conferring the discriminative binding of ATP analogs to cell-surface GRP78 over the purinergic receptors. The previously published synthesis protocols of ATP analogs with various 7-substituents, including halogen, carboxamide, alkyne, aryl, and heteroaryl groups [35–38] [39], offer a unique opportunity to accelerate the discovery of ATP analogs selective for GRP78_ATPase_.

## Conclusion

We have performed soaking experiments on GRP78_ATPase_ in the presence of ADP, ATP, and ATP analogs; presented the nucleotide-bound structures; and determined their relative binding affinities to GRP78_ATPase_. These data suggest that the replacement of the adenine nitrogen atom at the 7-position to a carbon atom does not affect the high-affinity binding to GRP78_ATPase_. However, the replacement of the triphosphate β-γ bridge oxygen with a carbon atom causes a reduction in GRP78_ATPase_ binding affinity. In addition, the 2’-OH group in the ribose sugar moiety is essential for the maintenance of high affinity binding to GRP78_ATPase_. This valuable information will aid the development of next-generation, ATP-based NTP analogs that contain modification(s) to confer metabolic stability against ecto-nucleotidases and avoid off-target effects while maintaining cytotoxicity against cells expressing cell-surface GRP78 (e.g., cancer cells).
