# Correction: Probing the ATP Site of GRP78 with Nucleotide Triphosphate Analogs

**DOI:** 10.1371/journal.pone.0158256

**Published:** 2016-06-21

**Authors:** Scott J Hughes, Tetyana Antoshchenko, Yun Chen, Hua Lu, Juan C Pizarro, Hee-Won Park

The following information is missing from the Funding section: Research described in this paper was in part performed by using the Biacore instrument, acquired with NIH Award (S10OD020117).
